# Fencing the genome: ubiquitin signaling restricts heterochromatin spread

**DOI:** 10.1038/s41392-025-02349-x

**Published:** 2025-08-21

**Authors:** Collin Bakker, Joanna Paulson, Nitika Taneja

**Affiliations:** 1 Department of Molecular Genetics, Erasmus University Medical Center, Erasmus MC Cancer Institute, Rotterdam, The Netherlands; 2 Oncode Institute, Rotterdam, The Netherlands

**Keywords:** Cell biology, Biochemistry, Epigenetics analysis

In a recent study published in *Science*, Tiebang Kang and his colleagues^1^ identify ASB7 as a key negative regulator of heterochromatin maintenance through targeted degradation of the H3K9me3 methyltransferase SUV39H1. The work uncovers a cell cycle-regulated chromatin-associated ubiquitin ligase circuit that maintains epigenetic homeostasis and reveals a novel vulnerability in cancer that may be exploited therapeutically.

H3K9me3 is a defining feature of constitutive heterochromatin, vital for transcriptional repression and genome stability. This repressive histone mark is propagated through a self-reinforcing loop, wherein HP1 binds pre-existing H3K9me3 and recruits SUV39H1 to methylate adjacent nucleosomes. While this mechanism ensures robust inheritance of silencing, unrestrained propagation could lead to widespread gene silencing and excessive chromatin compaction. How this feedback loop is constrained to maintain H3K9me3 homeostasis has remained poorly understood.

To uncover regulators that limit H3K9me3 spread, authors conducted a genome-wide CRISPR-Cas9 screen using H3K9me3 immunostaining in asynchronous and S-phase–arrested cells. Among known heterochromatin regulators such as SUV39H1/2, SETDB1, and EHMT1/2, the screen identified ASB7, a substrate adaptor of the CUL5 E3 ligase complex, as a top hit. ASB7 knockout led to genome-wide H3K9me3 accumulation across both repetitive and non-repetitive regions, confirmed by CUT&RUN and immunofluorescence. Conversely, ASB7 overexpression suppressed H3K9me3, supporting its role as a global repressor of heterochromatin expansion.

Mechanistically, ASB7 localizes to heterochromatin through interaction with HP1. Using TurboID-based proximity labeling and mass spectrometry, the authors mapped the ASB7 interactome, which included all HP1 isoforms and SUV39H1. ASB7 contains a conserved PxVxL motif within its ankyrin domain that mediates binding to the chromoshadow domain of HP1. Mutation of this motif disrupted the heterochromatin localization of ASB7 and diminished its repressive effect on H3K9me3, indicating that HP1 is essential for ASB7 recruitment.

Once localized to chromatin, ASB7 targets SUV39H1 for proteasomal degradation. Proteomic analysis showed that ASB7 overexpression specifically reduced SUV39H1 levels. This was validated across cell lines and DepMap data, which revealed an inverse correlation between ASB7 and SUV39H1 abundance. In vitro assays confirmed ASB7-mediated ubiquitylation of SUV39H1 at lysine 138. Notably, SOCS domain of ASB7 facilitated oligomerization and enhanced E3 ligase activity, suggesting a multivalent mechanism of substrate recognition.

Importantly, the authors showed that ASB7 function is further modulated by cell cycle cues. During mitosis, CDK1–Cyclin B1 phosphorylates ASB7 at T119, T152, and T216, disrupting its ability to bind and degrade SUV39H1. Phosphomimetic ASB7 mutants failed to localize to chromatin or reduce SUV39H1 levels, whereas phospho-dead mutants retained full activity. This mitotic inactivation of ASB7 permits transient SUV39H1 accumulation and H3K9me3 re-establishment post-replication, reminiscent of the concentration-dependent Clr4 spreading seen in fission yeast.^2^ ASB7 is reactivated in G1, restricting further H3K9me3 propagation.

However, it remains unclear how H3K9me3 spreading remains confined to heterochromatin domains during periods when ASB7 is transiently inactivated, such as mitosis in wild-type cells. Notably, in ASB7-deficient cells, SUV39H1 and H3K9me3 invade and appear within euchromatic regions, indicating that ASB7 is a dominant interphase regulator restricting heterochromatin expansion. These observations suggest that additional genomic or architectural mechanisms may contribute to maintaining epigenomic compartmentalization when ASB7 is inactive.^3^

To investigate clinical relevance, the authors analyzed TCGA data, which revealed truncating mutations in ASB7, particularly in its SOCS domain, impairing its E3 ligase function. CRISPR-engineered mouse models carrying these mutations exhibited increased SUV39H1 and H3K9me3 levels. Further, ASB7 overexpression impaired homologous recombination (HR) repair without affecting non-homologous end joining (NHEJ), and sensitized cells to PARP inhibitors such as Olaparib. While xenograft models showed that ASB7-overexpressing tumors grew more slowly and responded better to PARPi, the study did not provide direct evidence for altered H3K9me3 levels in response to DNA damage or PARPi treatment. Instead, the synthetic lethal interaction was inferred from the rescue effect of SUV39H1 co-expression, leaving open the possibility that ASB7 may influence HR through mechanisms independent of damage-associated H3K9me3, potentially via transcriptional repression or regulation of HR-related factors.

Whether ASB7 directly regulates chromatin at DNA damage sites remains an open question. While its recruitment to constitutive heterochromatin via HP1 is well established, it is unclear whether ASB7 also localizes to facultative heterochromatin, such as that formed in response to oncogene activation, metabolic stress, or within stress-induced open DSB-associated chromatin compartments.^4^ Recruitment mechanisms independent of HP1, potentially mediated by post-translational modifications or DNA damage checkpoint signaling, could broaden role of ASB7 in genome surveillance.

Further questions arise in the context of replication stress. PARP inhibitors are known to induce replication stress, and recent studies have shown that SUV39H1, in cooperation with G9a(EHMT2), catalyzes de novo H3K9me3 deposition at stalled replication forks to promote fork stability. Loss of SUV39H1 under these conditions results in fork degradation and elevated DNA damage, impairing genome-wide fork recovery.^5^ These observations raise the possibility that ASB7-mediated SUV39H1 depletion during replication stress may similarly destabilize forks, contributing to the observed sensitivity to PARP inhibitors. Whether ASB7 is dynamically recruited to stalled forks or fork-associated breaks in these contexts remains an open question.

Additionally, while the authors ruled out a major role for KDM4 demethylases in steady-state H3K9me3 regulation, the potential contribution of KDM3 family demethylases, which act on H3K9me1 and me2, remains unexplored in these settings. Given that SUV39H1 methylates H3K9me2 to generate H3K9me3, upstream removal of mono- and di-methyl marks by KDM3 enzymes could limit substrate availability and more profoundly affect H3K9me3 levels than KDM4-mediated demethylation alone. Previous studies have linked KDM3A/B to heterochromatin disassembly^3^ and replication-associated chromatin remodeling,^5^ suggesting a possible intersection with ASB7–SUV39H1 dynamics that may merit further investigation.

**Fig. 1:**
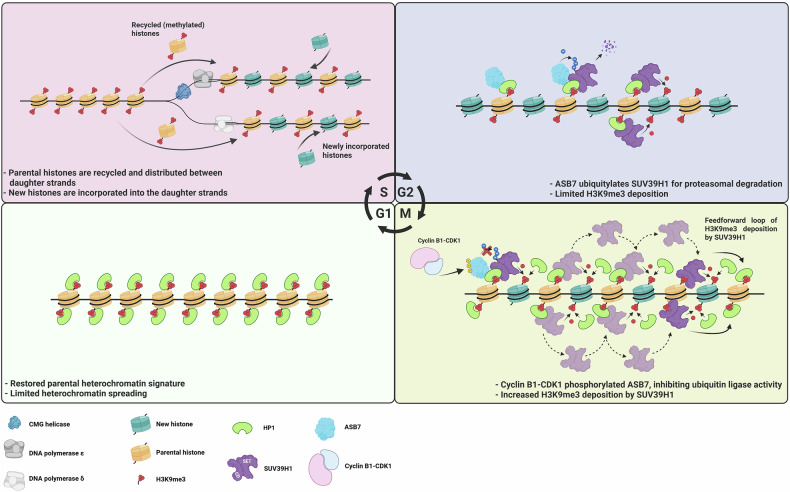
The HP1–SUV39H1–ASB7 regulatory loop orchestrates H3K9me3 homeostasis across the cell cycle. During S-phase (top left), parental H3K9me3-marked histones are redistributed between daughter strands, and incorporation of newly synthesized histones leads to dilution of the repressive mark. To counteract excessive H3K9me3 spreading driven by the HP1–SUV39H1 positive-feedback loop (top right), ASB7 ubiquitylates chromatin-bound SUV39H1, targeting it for proteasomal degradation and thereby restricting its invasion into euchromatic regions. In mitosis (bottom right), CDK1–Cyclin B1 phosphorylates ASB7, transiently inhibiting its E3 ligase activity and permitting SUV39H1 accumulation and H3K9me3 re-establishment on new histones. Following mitosis (bottom left), ASB7 is reactivated to restrict further spreading, thereby ensuring restoration and maintenance of heterochromatin identity across cell divisions. Created in Biorender (https://BioRender.com)

In summary, this study uncovers ASB7 as a pivotal chromatin-bound E3 ligase that restrains SUV39H1 to maintain heterochromatin homeostasis and prevent its aberrant spread (Fig. 1). By coupling activity of ASB7 to the cell cycle and linking its loss to heightened sensitivity to PARP inhibitors, the authors delineate a dynamic regulatory axis that safeguards genome integrity. These findings position ASB7 not only as a critical enforcer of heterochromatin boundaries, but also as a promising biomarker for predicting therapeutic response in cancers with disrupted epigenetic landscapes.
